# Persistent Conjunctival Chemosis after Lower Lid Blepharoplasty: A Comparison of Different Surgical Techniques

**DOI:** 10.3390/jcm13072093

**Published:** 2024-04-03

**Authors:** Alessandra Di Maria, Gianmaria Barone, Alessandro Gaeta, Filippo Confalonieri, Paolo Vinciguerra, Valeriano Vinci, Marco Klinger, Vanessa Ferraro

**Affiliations:** 1 Department of Ophthalmology, IRCCS Humanitas Research Hospital, Rozzano, 20089 Milan, Italy; alessandra.di_maria@humanitas.it (A.D.M.); gianmaria.barone@humanitas.it (G.B.); filippo.confalonieri@humanitas.it (F.C.); paolo.vinciguerra@hunimed.eu (P.V.); 2 Department of Biomedical Sciences, Humanitas University, Pieve Emanuele, 20072 Milan, Italy; 3 Department of Internal Medicine and Medical Specialties (DIMI), Università di Genova, 16132 Genova, Italy; alessandrogaeta01@gmail.com; 4 Plastic Surgery Unit, IRCCS Humanitas Research Hospital, Rozzano, 20089 Milan, Italy; marco.klinger@humanitas.it; 5 Department of Medical Biotechnology and Translational Medicine BIOMETRA, Reconstructive and Aesthetic Plastic Surgery School, University of Milan, 20089 Milan, Italy

**Keywords:** blepharoplasty, chemosis, oculoplastics, eyelid disease, eyelid surgery

## Abstract

**Background:** Conjunctival chemosis, a complication of lower blepharoplasty, can cause persistent discomfort and functional disturbances with worsening in the postoperative period following surgery. **Methods:** A review of the records of the lower blepharoplasty procedures carried out at the Humanitas Research Hospital, Rozzano, Milan, Italy was performed. Patients were categorized into two groups depending on the procedure performed: (1) transconjunctival blepharoplasty with the removal of the fatty lodges with canthopexy and (2) transcutaneous blepharoplasty with the removal of the fatty lodges with lateral canthoplasty. Each group was further divided into two more groups based on the surgical method used, that is either (a) cold blade and disposable cautery or (b) radiofrequency cut and coagulation and colorado tip (respectively 1a, 1b, 2a and 2b). All patients underwent a postoperative follow-up up to 24 months, which included an evaluation of cosmetic appearance, eyelid scarring and the severity of chemosis. The aim of the study was to investigate which of the surgical procedures causes a lower incidence of persistent type 3 conjunctival chemosis. **Results:** A total of 1047 patients who underwent lower lid blepharoplasty were included in the study. A total of 512 patients underwent transcutaneous blepharoplasty and 535 underwent the transconjunctival procedure. Among the first group of patients, 266 belong to group 1a and 246 to group 1b. In the second group, 264 were categorized as group 2a and 271 as group 2b. The incidence of type 3 chemosis in the transcutaneous blepharoplasty procedure with lateral canthoplasty was statistically significantly higher than in the transconjunctival approach, considering both the cold blade and the radiofrequency (*p* = 0.012, 0.010, 0.006, 0.004, respectively). **Conclusions:** A higher incidence of persistent type 3 conjunctival chemosis is associated with lateral canthus surgery and with the use of radiofrequency.

## 1. Introduction

Lower lid blepharoplasty, a surgical procedure aimed at rejuvenating the appearance of the lower eyelid region/cheek complex, has undergone significant advancements over the years [1]. The periorbital region demands meticulous surgical techniques to achieve both aesthetic and functional outcomes [2]. The lower eyelid region is characterized by complex anatomical structures, including the orbicularis oculi muscle, the orbital septum, the retaining ligaments and the underlying fat compartments. A disruption of any of these structures can lead to functional and aesthetic complications [3].

Traditional lower lid blepharoplasty techniques, such as the transcutaneous and transconjunctival approaches, have served as the foundation for periorbital rejuvenation. The transcutaneous approach involves an external incision made along the lower eyelid margin, allowing for a direct visualization and excision of excess skin and fat. Conversely, the transconjunctival approach utilizes a conjunctival incision to access and reposition orbital fat while avoiding visible external scars. The choice between these two approaches depends on the patient’s specific needs and anatomy. Transcutaneous blepharoplasty may be preferred for patients with significant skin laxity or when skin removal is necessary. Transconjunctival blepharoplasty, on the other hand, is suitable for those primarily concerned with fat pad removal and minimal skin excess [4]. Ultimately, the decision should be made through a careful assessment of the patient’s individual goals and anatomical characteristics to achieve the best aesthetic outcome. Moreover, the surgery of the lower lid can be associated with surgical interventions targeting the canthal region of the eye, in order to reshape the lateral canthus. Canthoplasty is a more invasive procedure that involves the manipulation and repositioning of the lateral canthal tendon, typically by detaching and reattaching it to a new location on the orbital bone. This method allows for a significant alteration of the eyelid’s shape or position [5]. Conversely, canthopexy is a less invasive technique that aims to provide support and stability to the canthal structures without tendon repositioning. It involves reinforcing the existing attachments of the lateral canthus, often utilizing sutures or grafts to augment the support without significantly altering the position [6].

Complications can range from minor and transient to more severe and lasting, underscoring the importance of surgical precision and patient selection. Among these, one of the minor complications of this surgical treatment, and in particular of lower blepharoplasty, is acute or chronic conjunctival chemosis [7].

Chemosis is defined as a transudative edema of the bulbar conjunctiva and/or fornix that creates a space between the conjunctiva and Tenon’s capsule and is characterized by a visible elevation of the conjunctival tissue with an underlying straw-colored fluid collection [8].

According to published data, the incidence of chemosis is about 1%. However, with recent improvements in surgical approaches, such as the shift from traditional, basic skin straightening and tightening procedures like blepharoplasty and face lifts to more involved treatments like lateral canthal support and orbicularis surgery, the problem has grown. Published rates of chemosis complications that last for two or three weeks have increased from 1% to 34.5% [9,10].

The purpose of this study is to understand which surgical procedure has a lower incidence of persistent conjunctival chemosis.

## 2. Materials and Methods

A retrospective review of the medical records of lower blepharoplasty procedures was performed. Patients who underwent primary blepharoplasty with a correction of the lateral canthus with cantoplasty or canthopexy were included in the study. We excluded patients affected by dysthyroidism, conjunctivocalasis and with a history of allergy or atopia. The study adhered to the guidelines of the Health Insurance Portability and Accountability Act and aligned with the principles set forth in the Declaration of Helsinki (2013). Patient consent was secured for the publication of the medical photographs featured in the article. Furthermore, the Institutional Review Board granted approval for the study. All of the patients included were divided into two groups as follows:

1. The first group underwent a transconjunctival procedure with a removal of the fatty lodges with canthopexy.
2. The second group underwent a transcutaneous blepharoplasty with a removal of the fatty lodges with lateral canthoplasty.

Each group was further classified on the basis of the instrumentation and material used:

- Cold blade and disposable cautery + fibrillar tabotamp and cold water irrigation.
- Radiofrequency cutting and coagulation (Surgitron Dual Frequency 120 Watts, Ellman International Inc., Hewlett, NY, USA; a high frequency/low temperature radiosurgical machine operating at 4.00 MHz). Cut mode: power: 30–35, using an A-8 needle for cutting and incision, cold-water irrigation and the Empire needle for the dissection and excision of skin (muscle) flaps in the coagulation mode (power: 35–40).

Under the guidance of an expert surgeon, every surgery was performed. Under local anesthetic, mebipuvacaine was injected for a regional block and lidocaine with epinephrine at a 1:100,000 concentration was injected locally for the incision. The patient was sedated with a gradual intravenous infusion of 2 mL of fentanyl chlorhydrate and 3 mg of midazolam after heart and peripheral oximetry were checked. Then, 10% betadine was used for skin disinfection and 5% for cleansing the conjunctival arches for 3 min. During the procedure the ocular surface was irrigated with isotonic solution and the cornea protected with viscoelastics.

**Figure 1:**
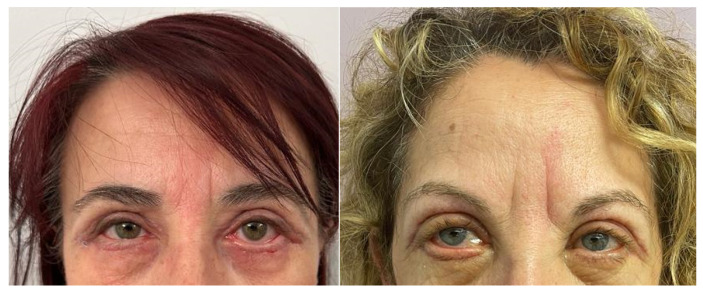
Two cases of lower lid blepharoplasty performed with subciliary skin incision 2 mm below the eyelash line.

The technique performed in transcutaneous lower blepharoplasty involves the execution of a subciliary skin incision 2 mm below the eyelash line, from which the underlying structures were reached. Following the release of the orbito-malar ligament, a lower eyelid cutaneous muscle flap was performed. This was followed by the transposition or removal of fat and a cantoplasty to treat the laxity of the tarsus ligament. (Figure 1). The orbicularis oculi muscle is cut, then the lateral canthal tendon is separated from the bone (cantholysis) and shortened. The tarsus is then moved to cover the lateral orbital rim and reattach it at the appropriate height. After seven days, the divided 6-0 nylon sutures used to seal the incisions were taken out.

In transconjunctival blepharoplasty, the placement of the transconjunctival incision is approximately 8 mm below the lid margin, allowing for transconjunctival access to the orbital fat. No suturing of the conjunctival incision was performed and lateral canthopexy was performed instead to avoid the so called “round eye” and to correct the lid length to compensate for the vertical volume reduction inherent in fat removal. The canthal tendon was exposed by a breach in the orbital septum that began at the inner edge of the lateral orbital bone and lined up with the outermost portions of the eyes. A precise incision was made above and below the tendon to allow a Prolene 4-0 suture needle to pass beneath it. The eye will be sutured to the periosteum of the upper lateral portion of the outer orbital wall once its form and retraction have been adjusted to the desired extent. Following surgery, patients received ointment, eye drops, and extensive corneal hydration along with eye protection.

All patients underwent postoperative follow-up at 7 days, 15 days and 1, 3, 6, 9, 12 and 24 months. During the assessment the classification of chemosis proposed by AB Weifeld et al. [11] (Table 1) was used to classify the results. 

Type 1 chemosis is an acute chemosis that is moderate. One unique characteristic is the capacity to fully close the eyelid.

Severe acute chemosis is represented by type 2 chemosis. A mass effect is created when the injected and enlarged conjunctiva protrudes from the palpebral opening. Chemosis-induced lagophthalmos is the ultimate outcome.

Type 3 chemosis is characterized by subchronic, ongoing conjunctival inflammation and edema that can last up to six months. The chronic lymphatic dysfunction brought on by insufficient lymphatic channel division and absence of re-anastomosis inside the eyelids and surrounding face tissues is most likely the cause of type 3 chemosis. In most cases, lagophthalmos is absent. 

Chemosis resulting from ectropion and/or a malposition of the lower eyelid is referred to as type 4 chemosis. It is critical to differentiate lagophthalmos brought on by the mass impact of severe chemosis in type 2 from the related partial lid closure in type 4. Type 4 results from conjunctival exposure and has both edema and inflammation.

Until the ectropion and malposition of the lower eyelids are rectified, type 4 chemosis will not resolve.

For the statistical analysis StataSE 18 software (StataSE, College Station, TX, USA) was used. A chi-square test was performed to measure the significant differences among the four groups. The level of significance was set at *p* < 0.05 and measured between groups 1a and 1b, 2a and 2b, 1a and 2a, and 1b and 2b.

## 3. Results

**Figure 2:**
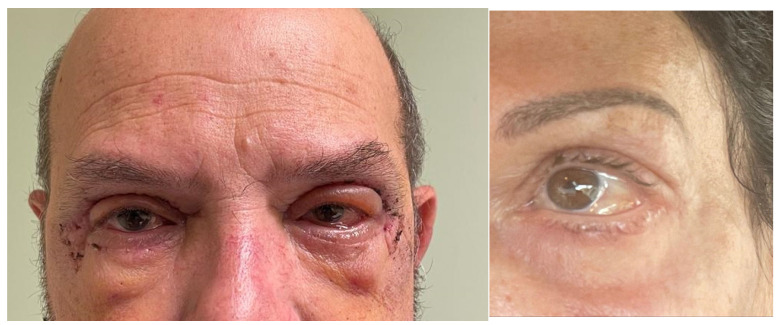
Conjunctival chemosis. On the left, an example of type 1 acute conjunctival chemosis associated with superior lid edema in a patient who underwent transcutaneous blepharoplasty. On the right, an example of type 3 persistent chemosis.

A total of 1047 patients who underwent lower lid blepharoplasty between 2005 and 2023 at the Department of Ophthalmology, Humanitas Research Hospital, Rozzano, Milan, Italy were included in the study. The majority of patients, 785 (74.9%) were female, while 262 (25.1%) were male. The total mean age was 58.3 ± 8.7 years old; the female cohort was 54.7 ± 9.4 and the male cohort was 69.7 ± 5.4. A total of 512 patients underwent transcutaneous blepharoplasty with lateral canthoplasty and 535 underwent a transconjunctival procedure with canthopexy. Among the first group of patients, 266 received treatment with cold blade and disposable cautery and 246 were treated with radiofrequency cut and coagulation and colorado tip. In the second group, 264 were treated with the cold blade technique and 271 with the radiofrequency technique (Figure 2). The average time taken to perform a bilateral procedure was 48 min for transconjunctival and 56 for transcutaneous. 

The characteristics of the sample are summarized in Table 2.

The mean follow-up time was 27.8 ± 2.3 months.

The main outcome was to measure the incidence of persistent type 3 conjunctival chemosis following different techniques of lower eyelid blepharoplasty and different materials and methods to perform them.

The incidence of postoperative conjunctival chemosis, considering the four groups of patients, is summarized in Table 3. 

Table 4 displays the *p*-values resulting from the analysis of the coupling of the four groups.

No significant surgical complications occurred. Certain issues that have been documented in the literature, including changes in eyelid closure, lagophthalmos, epicanthal, eyelid ptosis, drooping eyebrows, senile orbit and diplopia, were not observed [12,13].

## 4. Discussion

Transudative edema of the bulbar conjunctiva and/or fornix is known as conjunctival chemosis, and it is characterized by the conjunctiva visibly enlarging. It is a collection of fluid in the space between the capsule of the tenon that covers the sclera and the bulbar conjunctiva. Conjunctival chemosis manifests as epiphora, a sensation of a foreign body, because the conjunctiva is exposed, de-epithelialization occurs because of the exposure to hyperemia, and mild vision abnormalities occur because of the tear film’s abnormal distribution [14,15,16].

Chemosis generally does not result in a very disabling consequence; however, it can induce irritability, epiphora, and mild changes in visual acuity because of changes in the tear film’s distribution. Irregularities on the ocular surface can lead to epiphora as well as corneal dryness by interfering with the tear film’s natural lamination and drainage. This may undoubtedly make patients less satisfied with how well their surgery turned out cosmetically. Chemosis usually disappears on its own in the early postoperative phase, although in certain patients, remission takes longer. The aim of our study was to study whether a difference in the incidence of type 3 chemosis exists among four groups of patients treated with different surgical techniques.

The principal finding of our investigation was a statistically significant variation in the incidence of type 3 chemosis, with a higher incidence in transcutaneous blepharoplasty involving lateral canthus correction than in transconjunctival blepharoplasty, as well as a higher incidence when radiofrequency is employed.

The etiology of conjunctival chemosis following eyelid surgery is complex and usually involves a confluence of factors including inflammation, vascular permeability, lymphatic drainage impairment, and venous congestion. Thus, discomfort from the cleaning regimen, intraoperative conjunctival exposure, decreased eyelid tone during the postoperative phase, and inadequate lymphatic drainage as a result of dissection and healing are all thought to be the causes of chemosis [17,18].

The rates of published complications of chemosis persisting beyond 2 or 3 weeks have increased from 1% to 34.5%, which is paradoxical given the advancements in surgical techniques related to blepharoplasty and upper third face lift. These techniques combine the treatment of lateral canthal reinforcement and the dissection of the orbicularis muscle with maintaining ligament division and correcting for the upper half of the cheek [18,19].

Conjunctival inflammation is commonly observed in conjunction with type 1 chemosis, suggesting that venous engorgement and enhanced vascular permeability are the causes of extravasation. 

Our findings are consistent with the pathogenesis of type 1 chemosis, as no statistically significant difference was found among the four groups, except for the comparison between group 2a and 2b. 

On the contrary, type 3 chemosis is not supported by inflammatory processes, the conjunctiva is not hyperemic and this indicates the failure of lymphatic drainage as the main cause [20,21].

Although conjunctival inflammation is noted to be induced during the preoperative period by the disinfection regimen using polyvinylpyrrolidone-iodine (PVP-I), a broad-spectrum biocompatible antibacterial, this molecule is utilized in daily clinical practice without causing substantial side effects. 

It is known in the literature that concentrations equal to or lower than 0.5% of PVP-I are practically non-irritating even if administered up to six times a day. This protocol is used routinely in bulb surgery where there is no evidence of conjunctival chemosis and this leads to the exclusion of antiseptic treatment as a cause of chemosis [22,23].

The duration of surgery may induce proportional postoperative chemosis. Surgery time becomes longer when multiple procedures are combined. This may be due to long periods of exposure and dehydration with conjunctival and corneal de-epithelialization.

This consideration is mentioned in the literature, but not supported with specific studies [11,13].

The use of radiofrequency compared to the scalpel was evaluated in relation to the tissue damage and healing induced by the two surgical instruments because they may be involved in the manifestation of type 3 chemosis caused by the inflammation resulting from surgical trauma and the direct interruption of drainage lymphatic or indirect interruption due to scarring of the anatomical region treated.

There are not many studies that have taken these data into account, but the ones that have revealed no discernible differences between radiofrequency and scalpel incision in lower blepharoplasty in terms of scar formation, intratissue bleeding, inflammation, and the recovery of sensitivity [24]. Our findings are consistent with previous research [25,26].

Deep surgery (canthotomy and canthopexy) in the lateral canthal area, which can obstruct lymphatic drainage, was a common element in all of these treatments. 

It has long been known that the lateral region of the eyelids possesses a superficial collecting lymphatic system [9,10,27]. Dissection around the zygomatic-cutaneous ligaments and the orbicularis retention ligament may cause damage to the deep system, whereas incisions and dissection in the infraorbital area may cause harm to the superficial system. Therefore, injury to the superficial and deep lymphatic systems, especially on the lateral side, may be associated with prolonged edema and postoperative chemosis [28]. Chemosis usually disappears on its own in the early postoperative phase, although in certain patients, remission takes longer. Conjunctival chemosis can be treated in a variety of ways, including both conservative and surgical methods [17,29].

The conjunctiva and eyelids are drained by the deep lymphatic system, according to research done in 2016 by Shoukath et al. [28].

Prior histological and ink injection investigations have suggested the existence of a deep collecting lymphatic system [30,31,32,33].

The authors discovered that the superficial and deep facial lymphatic systems are connected to drain the lower eyelid and conjunctiva. In the infraorbital region, the superficial system is susceptible to injury during dissections and incisions. When the zygomatic-cutaneous and orbicularis retention ligaments are dissected, the deep system is susceptible to injury. Chemosis and prolonged edema may result from concurrent injury to the superficial and deep lymphatic systems, particularly laterally.

When surgical dissection and cauterization disturb preexisting conjunctival and cutaneous lymphatics, extracellular fluid may accumulate and produce conjunctival chemosis, lymphedema in the skin surrounding the eyelids, and subcutaneous abnormalities. In a similar vein, inadequate lymphatic drainage of the cornea and eyelids and a scarring of the lymphatic channels can result from severe or protracted postoperative inflammation. Conjunctival chemosis may also be caused by transient postoperative edema that causes localized venous congestion, even in the absence of injury to the nearby lymphatic channels.

As we previously discussed, the superficial lymphatic system is laterally dominating and drains the eyelids [10].

The preseptal orbicularis muscle allows the deep lymphatic system to join the superficial lymphatic system after draining the conjunctiva. Similar to the venous system of the head and neck, the lymphatic collecting veins are lacking in valves [34].

It has been found that the lymphatic pathways draining the face may also parallel the neurovascular system, with medial lymphatics following the facial vein and lateral lymphatics running adjacent to the facial nerve and vessels.

The conjunctiva and skin are drained by the superficial lymphatic system of the face, where precollectors (0.05 to 0.1 mm) form in the dermis close to the medial and lateral canthi. This form collects lymphatic vessels (>0.1 mm) at the level of the medial and lateral third of the orbicularis muscle retaining ligament, superficial to the preseptal orbicularis muscle. These gathering lymphatic veins move through the cheek’s subcutaneous fat. The lateral vessel is located in the lateral orbital fat compartment, and the medial vessel is located in the nasolabial fat compartment. Both of these vessels are still contained within their own fat compartments. The preauricular and submandibular nodes were the destinations of the lateral and medial collecting systems, respectively. Additionally, the precollectors go through the dermis and preseptal or bicularis muscle fibers to join the deep system, connecting the superficial and deep drainage systems.

Via the tarsal plate and meibomian glands in the lateral portion of the lower eyelid, the deep facial lymphatic system drains the conjunctiva straight from the precollectors. As previously indicated, linkages between the deep and facial lymphatic systems are made through the skin of the eyelid and face.

The deep system lymphatic precollectors then pass through the superficial orbicularis muscle retaining ligament and widen to reach the vessel collectors that travel in the fat sub-orbicularis oculi in the roof of the prezygomatic space. They pass beneath the surface of the preseptal orbicularis muscle in the lateral lower quadrant to the junction of the orbicularis muscle retaining ligament and the lateral orbital thickening. The collecting vessels descend into the preperiosteal fat, from which the zygomaticus major originates, at the level of the most cranial zygomaticocutaneous ligaments. From there, they travel beneath the deep fascia, beside the facial nerve, and eventually arrive at the preauricular lymph nodes within the parotid [28].

In our study, the incidence of type 3 chemosis was higher in blepharoplasty with the transcutaneous technique associated with canthoplasty compared to the transconjunctival technique associated with canthopexy.

A limitation of our study was the fact that no direct comparison between the pure transcutaneous and transconjunctival techniques is possible. In fact, the association between transcutaneous blepharoplasty and canthoplasty, as well as transconjunctival blepharoplasty and canthopexy, lies in their respective surgical approaches and goals.

Canthoplasty, which involves repositioning the canthal tendon and often requires access to deeper structures, is better facilitated through the same external incision utilized in transcutaneous blepharoplasty. In contrast, canthopexy aligns well with the less invasive nature of transconjunctival blepharoplasty, as both approaches involve minimal disruption to the external skin and focus on more conservative modifications. This association allows for a more harmonious and complementary surgical approach in addressing specific concerns of the eyelid region while minimizing potential scarring and preserving tissue integrity based on the depth of the incision. Hence, the comparison has to be considered between the procedures as a whole, as they involve the lateral canthus in different ways.

Of all of the factors considered, trauma to the lymphatic drainage system was the one that proved to be significant, as demonstrated by our results which show that the cases of chronic conjunctival chemosis were greater in transcutaneous surgery associated with canthoplasty compared to transconjunctival blepharoplasty associated with canthopexy. 

The results showed that the lower eyelid blepharoplasty technique with lateral canthus treatment performed by a single surgeon, with the same local and systemic anesthesia, with the use of the same bacteriostatic and the same postoperative therapy, has a higher incidence of type 3 chemosis with the transcutaneous technique compared to the transconjunctival one and that, in the two groups a and b which differ in the surgical materials used, group a of both techniques had a lower incidence of type 3 chemosis and faster resolution of acute post-surgical chemosis.

## 5. Conclusions

It is certain that for the prevention of type 3 chemosis all of the favorable factors discussed must be carefully considered, such as the use of bacteriostatics with known indications, abundant irrigation with washing solution and radiofrequency that respects parameters that do not histologically damage the tissues too much, both for cutting and hemostasis. The key point is the ‘judicious’ dissection of the lateral canthal structures in order to reduce direct or indirect interruption due to edema and the fibrotic reaction of the superficial and deep drainage systems, especially in transcutaneous surgery associated with canthoplasty where the incidence of persistent chemosis is higher in percentage than transconjunctival associated with canthopexy.

## Figures and Tables

**Table 1:** The classification of chemosis.

Class	Edema	Lagophthalmos	Duration
Type 1 (acute mild)	Mild	Absent	<3 wk
Type 2 (acute severe)	Severe	Present laterally (chemosis hinders closure)	>3 wk
Type 3 (subchronic)	Mild to severe	Absent	3 wk to 6 mo
Type 4 (subchronic because of lower lid malposition)	Severe	Lower lid malposition	Until lid malposition

**Table 2:** Characteristics of the sample.

N	1047
Sex (M)	262 (25.1%)
Sex (F)	785 (74.9%)
Total mean age	58.3 ± 8.7 years
Male mean age	69.7 ± 5.4
Female mean age	54.7 ± 9.4
Type of surgical procedure	
1. transconjunctival blepharoplasty with canthopexy cold blade and disposable cautery radiofrequency cut and coagulation and colorado tip	535 (51.1%) 264 271
2. transcutaneous blepharoplasty with lateral canthoplasty cold blade and disposable cautery radiofrequency cut and coagulation and colorado tip	512 (48.9%) 266 246

**Table 3:** Percentage of incidence of postoperative chemosis in the four groups of patients.

	Group 1a—264 Patients	Group 1b—271 Patients	Group 2a—266 Patients	Group 2b—246 Patients
Type 1 chemosis	58 (21.97%)	78 (28.78%)	65 (24.44%)	87 (35.37%)
Type 2 chemosis	24 (9.09%)	47 (17.34%)	39 (14.46%)	53 (21.54%)
Type 3 chemosis	1 (0.38%)	9 (3.32%)	10 (3.76%)	23 (9.35%)
Type 4 chemosis	0	0	0	0

**Table 4:** The *p*-values calculated between coupled groups of treatment.

	Group 1a x 1b	Group 2a x 2b	Group 1a x 2a	Group 1b x 2b
Type 1 chemosis	0.070	0.007	0.501	0.109
Type 2 chemosis	0.005	0.043	0.048	0.227
Type 3 chemosis	0.012	0.010	0.006	0.004

## Data Availability

The datasets generated during this study are available from the corresponding author upon reasonable request.
